# Porphyria cutanea tarda increases risk of hepatocellular carcinoma and premature death: a nationwide cohort study

**DOI:** 10.1186/s13023-019-1051-3

**Published:** 2019-04-03

**Authors:** Carl Michael Baravelli, Sverre Sandberg, Aasne Karine Aarsand, Mette Christophersen Tollånes

**Affiliations:** 1 Norwegian Porphyria Centre (NAPOS), Haukeland University Hospital, Bergen, Norway; 2 Department of Global Public Health and Primary Care, University of Bergen, Bergen, Norway; 3 Norwegian Quality Improvement of Laboratory Examinations (NOKLUS), Haraldsplass Deaconess Hospital, Bergen, Norway

**Keywords:** Porphyria cutanea tarda, Neoplasms, Liver neoplasms, Hepatocellular carcinoma, Gallbladder and bile duct neoplasms, Cause of death

## Abstract

**Background:**

Porphyria cutanea tarda (PCT) is a skin disorder originating from a deficit of the liver enzyme uroporphyrinogen decarboxylase. PCT may be a risk factor for hepatocellular carcinoma (HCC) and other cancers, but the evidence is unclear. We aimed to investigate cancer and premature mortality risk in persons with PCT.

**Methods:**

The cohort study consisted of all Norwegian residents from 18 years between 2000 and 2016 (*n* = 5.4 million). 612 persons with PCT, and all cancer diagnoses and causes of death were identified through record linkage between national registries. Hazard ratios (HRs) and corresponding 95% confidence intervals (CIs) were adjusted for age, sex, education and calendar years. We additionally compared persons with PCT to persons with a history of chronic alcohol abuse (*n* = 30,468).

**Results:**

Persons with PCT were more likely to be diagnosed with HCC [adjusted HR (aHR) = 19.7, CI = 8.8–44.0) and gallbladder and biliary tract cancer (aHR = 6.8, CI = 2.2–21.0) than the reference population. A moderate increased risk for HCC (aHR = 3.1, CI = 1.2–7.7) and gallbladder and biliary tract cancer (aHR = 4.0, CI = 1.1–14.4) remained when compared to persons with a history of chronic alcohol abuse. Additionally, compared to the reference population, persons with PCT had an increased risk of premature death (aHR = 1.5, CI = 1.2–1.7), due to the following causes of death: malignant neoplasms (aHR = 1.4, CI = 1.0–1.9), diseases of the liver (HR = 5.5, CI = 2.5–12.2), and drug and alcohol overdose (HR = 9.9, CI = 4.7–20.8).

**Conclusions:**

Persons with PCT had an increased risk of HCC and cancer of the gallbladder and biliary tract, as well as premature death. Although most of our findings can likely be explained by common lifestyle risk factors, something inherent in PCT may contribute to the development of HCC.

**Electronic supplementary material:**

The online version of this article (10.1186/s13023-019-1051-3) contains supplementary material, which is available to authorized users.

## Background

Porphyria cutanea tarda (PCT) results from a defect of hepatic enzyme uroporphyrinogen decarboxylase (UROD). The impaired UROD activity in the liver causes accumulation of uro and heptacarboxylated porphyrins, which act as photosensitizer in the skin and give symptoms in the form of bullae, fragile skin, hypertrichosis and hyperpigmentation [1]. PCT is the most prevalent form of porphyria worldwide, with symptom debut typically in middle age and an approximate equal sex ratio. In Norway, the prevalence is estimated at 1 in 10,000 [2]. PCT occurs in both an acquired (sporadic PCT) and a hereditary form (familial PCT) [3], which are clinically indistinguishable. Susceptibility factors for both include excess iron, hepatitis C and B infection, excessive alcohol intake, smoking, human immunodeficiency virus (HIV) and oestrogens [3–8], although susceptibility factors appear less strongly correlated with familial PCT [3, 6]. PCT is associated with hepatocellular carcinoma (HCC) [9–12], and persons with PCT may also have increased risk of premature death [12]. The main risk factors for HCC in the general population include Hepatitis C and B and an excessive alcohol intake [13, 14]. Whether PCT increases the risk for HCC over and above the risk caused by associated susceptibility factors is controversial. It also remains unclear whether the risk for HCC warrants HCC surveillance, as indicated for other types of hepatic porphyrias [15].

We aimed to investigate risks of cancer and premature mortality in persons with PCT compared to the general population, and to examine whether any increased risk was likely caused by PCT associated susceptibility factors or, in part, PCT itself.

## Methods

### Data sources

Data collection was derived from five nationwide Norwegian population based sources: The Norwegian Porphyria Registry [16], The Cancer Registry of Norway [17], The Cause of Death Registry of Norway [18], Statistics Norway, and the Norwegian Labour and Welfare Administration. Using a unique national 11-digit personal identification number, record linkage was performed in 2018.

Persons in Norway who are diagnosed with porphyria are invited to participate in the national Norwegian Porphyria Registry, administered by the Norwegian Porphyria Centre, established in 2000 [16]. Data is derived from patient-reported questionnaires supplemented with biochemical and genetic laboratory results. Data included in this report were updated one or two years after the first questionnaire, and thereafter every four years.

The mandatory national Norwegian Cancer Registry, records all new neoplasms since 1953. Diagnoses are based on a modified version of the 7th revision of the International Classification of Diseases (ICD-7), and completeness is greater than 98% [17]. The Cause of Death Registry of Norway has a coverage greater than 98% [18]. Diagnostic codes are based on ICD-9 and ICD-10.

Statistics Norway has maintained the Population Database, with demographic data for the entire population, since 1876, and The Norwegian Standard Classification of Education, with information about educational attainment, since 1970.

Statistics Norway and Norwegian Labour and Welfare Administration have maintained records regarding access to social security benefits: sick leave benefit, the disability pension, and medical and occupational - rehabilitation benefit since 1992. Diagnostic codes include the second revision of the International Classification of Primary Care (ICPC-2), ICD-9 and ICD-10.

### Study population

**Fig. 1:**
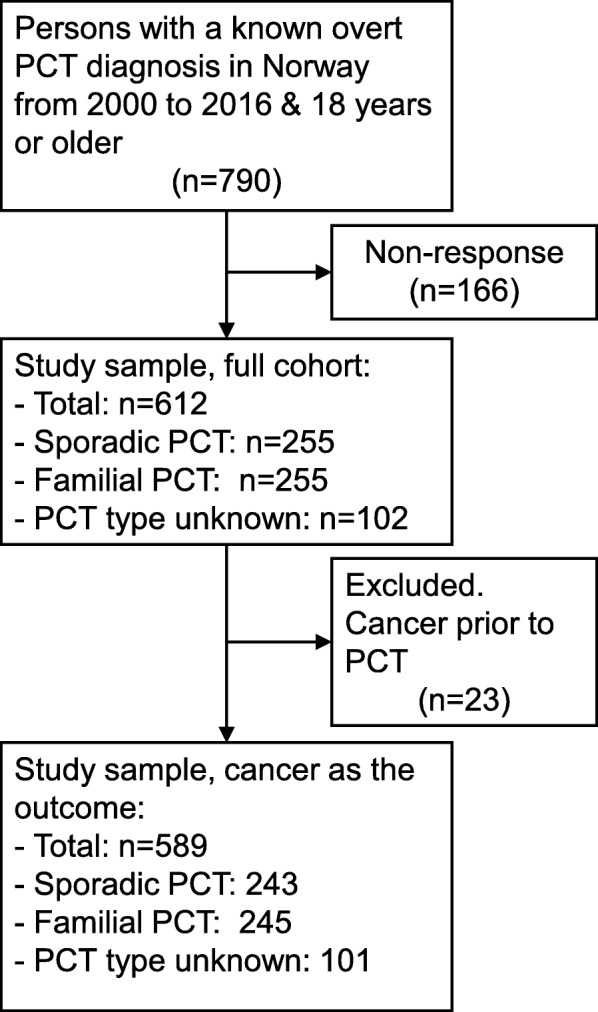
Overview of study sample

All Norwegian residents, registered in the Norwegian Population Registry and 18 years or older from 2000 to 2016 were included in our cohort, and the reference population comprised of 5,451,951 adults. Of the 790 persons with a known overt diagnosis of PCT in Norway, registered at the Norwegian Porphyria Centre, alive and aged 18 years or older between January 2000 and December 2016, 612 (78%) agreed to participate (Fig. 1). Where a cancer diagnosis was the outcome, we excluded 23 persons who had a cancer diagnosis proceeding PCT symptoms (Fig. 1).

PCT diagnosis was based on biochemical testing performed at the Department of Medical Biochemistry and Pharmacology, Haukeland University Hospital [3], applying diagnostic algorithms as described by Badminton and colleagues [19]. Urinary porphyrins were expressed in nmol/mmol creatinine. Sequencing of the *UROD* gene was performed at the Center for Medical Genetics and Molecular Medicine at Haukeland University Hospital to determine if the patient had familial or sporadic PCT. Participants where DNA analysis had not been performed, were registered as unclassified PCT.

For our sub-group analyses, we identified 30,468 persons with a diagnosis of chronic alcohol abuse/dependence through the Norwegian Labour and Welfare Administration registrations of social security benefits.

### Disease classification

Primary cancer diagnoses and underlying causes of death of theoretical interest, or with a count of three or greater, were investigated. This resulted in the following primary cancers: all sites (ICD-7: 140–207; ICD-10: C00–96, D45–47), colon/rectum (ICD-7: 153–154; ICD-10: C18-C21), HCC (ICD-7: 155.0; C22.0), gallbladder and biliary tract (ICD-7: 156; ICD-10:C23–24), pancreas (ICD-7: 157; ICD-10; C25), lung (ICD-7: 162; C33–34); non-melanoma skin (ICD-7: 191; ICD-10: C44), breast (ICD-7: 170; ICD-10: C50), prostate (ICD-7: 177; ICD-10: C61), and leukemia (ICD-9: 207; ICD-10: C91–95, D45–47). We included any new cases of primary cancer diagnosis from January 2000. We also assessed second primary cancers specifically. Similarly, the following underlying causes of death were investigated: malignant neoplasms (ICD-10: C00–96, D45–47), diabetes mellitus (ICD-10: E10–14), ischemic heart disease (ICD-10: I20–25), cerebrovascular diseases (ICD-10: I60-I69), chronic obstructive pulmonary disease (ICD-10: J43-J44), diseases of the liver (ICD-10: K70–77, B15–19, E83.1), renal failure (ICD-10: N17–19), and alcohol or drug overdose (ICD-10: X40–49). Chronic alcohol abuse/dependence was defined by the following codes: ICPC-2: P15; ICD-9: 303, 305.0; ICD-10: F10.

### Statistical analysis

Stata/SE Version 14 for Windows was used for all statistical analyses (StataCorp Stata Statistical, Software: Release 14, College Station, TX, USA). Annual incidence was calculated by dividing the number of new cases by the total person-years of all participants. The entry time was age at baseline or 18 if younger than 18 years in 2000 (i.e., left truncated). The exit time was age at event (cancer/mortality) or censoring (death due to other factors, or end of study follow-up in 2016, whichever occurred first). To assess the relationship between PCT and cancer/mortality, a Cox regression was used to estimate the hazard ratios (HRs) with 95% confidence intervals (CIs), in which PCT diagnosis (no/yes) was the exposure and the cancer or cause of death of interest (no/yes) the outcome. Where numbers permitted, we additionally investigated PCT type (0 = reference, 1 = sporadic PCT, 2 = familial PCT, 3 = PCT type unclassified PCT) as the exposure. Age was used as the time scale, stratifying the model by birth cohorts (< 1937, 1937–1960, 1961–1983, > 1983), to adjust for calendar effects. The multivariate models were adjusted for sex, age (as the time-scale), and educational attainment. The proportionality assumption was assessed and covariates entered as time-dependent when the assumption was violated. Obtained level of education was categorized into: no schooling, compulsory education (year 1–10), upper secondary education (11–13, or 14 years if including post-secondary non-tertiary education), tertiary education (14+ years), and unspecified/missing. Interactions of exposure by educational obtainment (tertiary or upper secondary vs less education) and by sex were explored, and none were found (although limited power for interaction analyses). Differences in age at time of death were investigated by independent samples t-tests.

For each adjusted hazard ratio (aHR), we calculated the corresponding evidence value (E-value). The E-value is defined as the minimum risk ratio an unmeasured confounder requires to explain away the outcome and is calculated by the formula: *E-value = hazard ratio (HR) + sqrt[HR x (HR-1)]* [20].

We compared cancer and causes of death risk in persons with PCT to persons with a history of chronic alcohol abuse/dependence, who have a high mortality risk. This was achieved by conducting a competing risks regression survival analysis calculating the adjusted subdistribution hazard ratio (aSHR). Persons with both PCT and a history of chronic alcohol abuse/dependence (*n* = 17) were excluded.

### Ethical approval

The study protocol conformed to the ethical guidelines of the 1975 Declaration of Helsinki and was approved by the Regional Committees for Medical and Health Research Ethics, Norway (reference number: 2012/753).

## Results

At entry into the study, persons with familial PCT, sporadic PCT and unclassified PCT were, on average, 9, 14 and 21 years older than the reference population, respectively. Additionally, persons with sporadic and unclassified PCT types, generally had less education and were more likely to smoke daily, and have type II diabetes than persons with familial PCT. However, there was no apparent difference regarding self-reported alcohol use (Table 1).

**Table 1:** Baseline characteristics of participants with sporadic (s-PCT), familial (f-PCT) and unclassified PCT compared to the reference population (2000 to 2016, 18 years or older)

Characteristics	s-PCT (*n* = 255)		f-PCT (*n* = 255)		Unclassified PCT (*n* = 102)		Reference (*n* = 5452,010)	
	n	Valid %	N	Valid %	n	Valid %	n	Valid %
Sex – male	125	49.0	122	47.9	46	45.1	2713,666	50.2
Age in years at study start (mean, SD)	52.8	(10.9)	47.5	(13.7)	60.1	(13.9)	38.9	(22.2)
Highest level of education obtained								
Not specified	4	1.6	2	0.8	0	0	649,456	11.9
No schooling, primary/middle education (year 1–10)	90	35.3	55	21.6	46	45.1	1334,399	24.8
Upper secondary education (11–12, 13 or 14 years)	124	48.6	123	48.2	43	42.2	1994,025	36.6
Tertiary education (14+ years)	37	14.5	75	29.4	13	12.8	1474,130	27.0
Lifestyle factors								
Alcohol use (yes)	163	93.6	173	93.5	35	87.5		
Alcohol – amount of standard drinks per week (mean, SD)	1.7	(1.3)	1.6	(1.3)	1.5	(1.3)		
Tobacco: cigarettes (yes)	83	32.6	50	19.6	30	61.2		
Never smoked / have quit	65	37.8	127	69.4	22	53.63		
Occasional smokers/ daily smokers	107	62.2	56	30.6	19	46.3		
Amount of cigarettes consumed per day (mean, SD)	2.8	(6.2)	1.2	(3.8)	1.7	(4.6)		
Body mass index (BMI)								
Underweight (BMI Under 18.5)	8	4.8	5	2.8	2	5.1		
Normal (BMI between 18.5 and 25)	67	40.4	56	30.9	13	33.3		
Overweight (BMI between 25 and 30)	68	41.0	90	49.7	21	53.8		
Obese (BMI of 30 or greater)	23	13.9	30	16.6	3	7.7		
Other diseases								
Liver disease	24	11.0	12	5.2	6	5.2		
Haemochromatosis	11	5.1	12	5.2	2	4.1		
Type II diabetes	11	5.1	3	1.3	2	7.4		
Biochemical characteristics								
Total porphyrins (mean, 95% CI)	880.5	803.6–957.5	941.6	835.7–1047.5	989.2	660.58–1317.81		
Uroporphyrin (mean, 95% CI)	608.5	553.5–663.4	706.1	625.03–787.1	768.45	504.01–1032.89		

Abbreviations: *PCT* porphyria cutanea tarda

Lifestyle factors are based on self-reported questionnaires sent to the Norwegian Porphyria Registry. 500 of 612 responded to the questionnaire. Biochemical characteristics are based on the highest ever recorded value (generally at time of diagnosis). Urinary porphyrins were expressed in nmol/mmol creatinine (upper reference limit < 30).

Persons with PCT had, on average, a 20-fold increased risk of HCC and an 7-fold increased risk of gallbladder and biliary tract cancer, compared to the general population. There was no evidence of an increased risk of all first primary cancers in persons with PCT, but we observed a 2-fold increased risk of all second primary cancer diagnoses. When assessed by PCT type (sporadic, familial, unclassified PCT), the risk was greatest in persons with unclassified PCT type (Table 2). The individual characteristics of persons with PCT and cancers are displayed in Additional file 1: Table S1.

**Table 2:** Annual incidence rates and hazard ratios for neoplasms in persons with porphyria cutanea tarda (2000–2016, 18 years or older)

Cancer codes (ICD-7; ICD-10)	Cancer cases/no. at risk	Person-years; annual incidence of cancer diagnosis per 100 (95% CI)	Hazard ratios (95% confidence intervals)		E-value
			Crude	Adjusted	
All sites – first primary C (140–207; C00–96, D45–47)					
Reference	358,701/5451951	70,185,262; 0.507 (0.506–0.509)	1.0	1.0	1.0
PCT (total)	80/589	8368; 0.956 (0.768–1.190)	1.1 (0.9–1.4)	1.1 (0.9–1.3)	1.4
Familial PCT	25/245	3728; 0.671 (0.453–0.992)	0.9 (0.6–1.4)	0.9 (0.6–1.3)	1.0
Sporadic PCT	34/243	3547; 0.959 (0.685–1.342)	1.1 (0.8–1.5)	1.0 (0.7–1.4)	1.1
Unclassified PCT	21/101	1094; 1.920 (1.252–2.945)	1.8 (1.1–2.7)	1.6 (1.0–2.5)	2.6
All sites – second primary C (140–207; C00–96, D45–47)					
Reference	57,505/5451951	71,305,121; 0.081 (0.080–0.081)	1.0	1.0	1.0
PCT (total)	26/589	8424; 0.309 (0.210–0.453)	2.4 (1.6–3.5)	2.2 (1.6–3.3)	3.8
Familial PCT	6/245	3749; 0.160 (0.72–0.356)	1.5 (0.7–3.3)	1.4 (0.6–3.1)	2.2
Sporadic PCT	14/243	3557; 0.394 (0.233–0.665)	2.9 (1.7–4.9)	2.8 (1.7–4.7)	5.0
Unclassified PCT	6/101	1119; 0.536 (0.241–1.194)	2.8 (1.2–6.1)	2.5 (1.1–5.6)	4.4
Colon/rectum (153–154; C18-C21)					
Reference	46,077/5451951	71,375,873; 0.503 (0.501–0.504)	1.0	1.0	1.0
PCT	11/578	8564; 0.936 (0.752–1.165)	1.2 (1.0–1.5)	1.1 (0.9–1.4)	1.4
Hepatocellular carcinoma (155.0; C22.0)					
Reference	1543/5451951	71,538,150; 0.002 (0.002–0.002)	1.0	1.0	1.0
PCT (total)	6/589	8567; 0.070 (0.031–0.156)	19.9 (8.9–44.3)	19.7 (8.8–44.0)	38.9
Gallbladder and biliary tract (156; C23-C24)					
Reference	1628/5451951	71,537,477; 0.003 (0.003–0.003)	1.0	1.0	1.0
PCT	3/589	8576; 0.035 (0.011–0.108)	7.2 (2.3–22.5)	6.8 (2.2–21.0)	13.0
Pancreas (157, C25)					
Reference	9830/5451951	71,375,873; 0.014 (0.014–0.014)	1.0	1.0	1.0
PCT	3/589	8546; 0.035 (0.011–0.109)	1.6 (0.5–4.9)	1.5 (0.5–4.6)	2.4
Lung (162, C33–34)					
Reference	34,920/5451951	71,498,290; 0.049 (0.048–0.049)	1.0	1.0	1.0
PCT	13/589	8571; 0.152 (0.088–0.261)	2.9 (1.7–5.0)	1.6 (0.9–2.8)	2.6
Non-melanoma skin (191, C44)					
Reference	16,048/5452,951	66,463,540; 0.024 (0.024–0.0024)	1.0	1.0	1.0
PCT	4/589	8533; 0.047 (0.018–0.125)	1.9 (0.7–5.1)	1.1 (0.4–3.5)	1.4
Breast (170; C50)					
Reference	39,581/2713314	35,414,769; 0.112 (0.111–0.113)	1.0	1.0	1.0
PCT	5/299	4337; 0115 (0.048–0.277)	0.6 (0.3–1.5)	0.6 (0.3–1.7)	1.0
Prostate (177; C61)					
Reference	56,652/2738637	35,644,567; 0.158 (0.157–0.159)	1.0	1.0	1.0
PCT	14/290	4170; 0.336 (0.199–0.567)	1.2 (0.7–2.0)	1.1 (0.6–1.8)	1.4
Leukaemia (207; C91–95, D45–47)					
Reference	16,349/5451951	72,014,044; 0.023 (0.022–0.023)	1.0	1.0	1.0
PCT	4/589	8560; 0.023 (0.018–0.125)	1.4 (0.5–3.7)	1.3 (0.5–3.5)	1.1

Crude analysis adjusted for age as the time-scale. Adjusted analysis covariates include age as time-scale, sex (except for gender specific cancers), educational obtainment, and birth cohorts.

Note that 23 persons diagnosed with cancer prior to their first reported PCT symptoms or diagnosis were excluded. Diagnosis based on the International Classification of Diseases, 7th (ICD-7) and 10th revision (ICD-10) codes.

The mean age at death for persons with PCT was 72.2 years (95% CI: 70.3–74.1) compared to 78.5 years (95% CI: 78.4–78.5) in the general population (mean difference = 6.3, 95% CI: 4.4–8.2, *p* < .001). There was no detectable difference in the mean age at death between persons with sporadic PCT (mean = 71.8; 95% CI: 68.9–74.7), familial PCT (mean = 72.1; 95% CI: 67.0–77.1) and unclassified PCT type (mean = 73.3; 95% CI: 70.5–76.1).

Compared to the general population, persons with PCT had, on average, a 50% increased risk of premature death, 40% increased risk of death by malignant neoplasms (all-sites), a 6-fold excess risk of death by liver diseases, and a 10-fold excess risk of death by alcohol or drug overdose. The risk of premature death and death by malignancies was highest in persons with unclassified PCT (Table 3).

**Table 3:** Annual mortality rates and hazard ratios for causes of death in persons with porphyria cutanea tarda (2000–2016, 18 years or older)

Sample	Mortality cases/no. at risk	Person-years; annual mortality rate per 100 (95% CI)	Hazard ratios (95% confidence intervals)		E-value
			Crude	Adjusted	
Premature death					
Reference	748,900/5451,951	71,559,947; 1.047 (1.044–1.049)	1.0	1.0	1.0
PCT (total)	158/612	8921; 1.771 (1.515–2.070)	1.5 (1.3–1.8)	1.5 (1.2–1.7)	2.4
Familial PCT	27/255	3933; 0.686 (0.471–1.001)	0.7 (0.5–1.1)	0.8 (0.1–1.1)	1.0
Sporadic PCT	64/255	3828; 1.672 (1.309–2.136)	1.5 (1.2–1.9)	1.4 (1.1–1.8)	2.2
Unclassified PCT	67/102	1160; 5.776 (4.546–7.339)	2.6 (2.1–3-4)	2.5 (1.9–3.1)	4.4
Malignant neoplasms (C00–96)					
Reference	181,205/5,451,951	71,559,947; 0.254 (0.253–0.255)	1.0	1.0	1.0
PCT (total)	48/612	8921; 0.538 (0.405–0.714)	1.4 (1.1–1.9)	1.4 (1.0–1.9)	2.2
Sporadic PCT	23/255	3828; 0.601 (0.399–0.904)	1.6 (1.0–2.4)	1.5 (1.0–2.2)	2.4
Familial PCT	11/255	3933; 0.280 (0.155–0.505)	0.9 (0.5–1.7)	0.9 (0.5–1.6)	1.0
Unclassified PCT	14/102	1160; 1.207 (0.715–2.038)	2.0 (1.2–3.4)	1.8 (1.1–3.1)	3.3
Diabetes mellitus (E10–14)					
Reference	11,826/5452,010	71,559,947; 0.016 (0.016–0.017)	1.0	1.0	1.0
PCT	3/612	8921; 0.034 (0.011–0.104)	2.0 (0.7–6.3)	1.7 (0.5–5.3)	2.8
Ischemic heart disease (I20–25)					
Reference	5,451,951	71,559,947; 0.135 (0.134–0.136)	1.0	1.0	1.0
PCT	7/612	8921; 0.079 (0.037–0.165)	0.6 (0.3–1.2)	0.6 (0.3–1.2)	1.0
Cerebrovascular diseases (I60-I69)					
Reference	57,639/5,451,951	71,559,947; 0.081 (0.080–0.081)	1.0	1.0	1.0
PCT	8/612	8915; 0.090 (0.045–0.179)	1.2 (0.6–2.4)	1.1 (0.6–2.3)	1.4
Chronic obstructive pulmonary disease (J43–44)					
Reference	31,172/5,451,951	71,559,947; 0.043 (0.043–0.044)	1.0	1.0	1.0
PCT	7/612	8921; 0.078 (0.037–0.165)	1.4 (0.6–2.9)	1.4 (0.6–2.8)	2.2
Diseases of the liver (K70–77, B15–19, E83.1) ^a^					
Reference	4661/5,452,010	71,559,947; 0.007 (0.00–0.007)	1.0	1.0	1.0
PCT	6/612	8921; 0.067 (0.030–0.150)	5.9 (2.7–13.2)	5.5 (2.5–12.2)	10.5
Renal failure (N17–19)					
Reference	6686/5,452,010	71,559,947; 0.009 (0.009–0.010)	1.0	1.0	1.0
PCT	2/612	8921; 0.022 (0.006–0.090)	2.9 (0.7–11.8)	2.9 (0.7–11.7)	5.3
Drug and alcohol overdose (X40–49) ^b^					
Reference	4961/5452,010	71,559,947; 0.007 (0.007–0.007)	1.0	1.0	1.0
PCT	7/612	8921; 0.078 (0.037–0.165)	12.1 (5.8–25.5)	9.9 (4.7–20.8)	19.3

Disease codes defined using the International Statistical Classification of Diseases 10th (ICD-10) revision

^a^Specific diagnoses of the 6 PCT patients with liver disease were: alcoholic cirrhosis of liver (K70.3), alcohol hepatic failure (K70.4) primary biliary cirrhosis (K74.3), hepatic failure (K72.9), 2 x chronic viral hepatitis C (B18.2). Familial PCT, *n*=0, sporadic PCT, *n*=2, Not tested, *n*=3. None of the six persons with liver disease were diagnosed with HCC or gallbladder and biliary tract cancer.

^b^Specific diagnosis of the 7 persons with PCT and drug and alcohol overdose included: 4 x accidental poisoning by and exposure to narcotics and psychodysleptics [hallucinogens], not elsewhere classified (ICD-10: X42); 1 x accidental poisoning by and exposure to non-opioid analgesics, antipyretics and antirheumatics (ICD-10: X40); 1 x accidental poisoning by and exposure to antiepileptic, sedative-hypnotic, antiparkinsonism and psychotropic drugs, not elsewhere classified (ICD-10: X41); 1 x accidental poisoning by and exposure to alcohol (X45). Familial PCT, *n*=1, sporadic PCT, *n*=3, Not tested, *n*=3

Compared to persons with a history of chronic alcohol abuse/dependence, persons with PCT were at a 3-fold increased risk of HCC and 4-fold increased risk of gallbladder and biliary tract cancer, and had an 80% reduced risk of dying from non-malignant liver diseases (Table 4).

**Table 4:** Annual incidence/mortality rates and subdistribution hazard ratios for malignancies and causes of death in persons with porphyria cutanea tarda (2000–2016, 18 years or older)

	Incidence cases/no. at risk	Person-years; annual mortality rate per 100 (95% CI)	Subdistribution hazard ratios (95% confidence intervals)	
			Crude SHR (95% CI)	Adjusted SHR (95% CI)
All sites – first primary C (C00–96, D45–47)				
Reference	3549/30468	393,347; 0.902 (0.873–0.932)	1.0	1.0
PCT	78/572	8132; 0.959 (0.768–1.198)	0.7 (0.5–0.8)	0.7 (0.6–0.9)
HCC (155.0; C22.0)				
Reference	80/30,468	402,342; 0.020; 0.020 (0.16–0.025)	1.0	1.0
PCT	5/572	8330; 0.060 (0.025–0.144)	2.2 (0.9–5.5)	3.1 (1.2–7.7)
Gallbladder & biliary tract (156; C23–24)				
Reference	19/30,468	402,403; 0.003 (0.003–0.007)	1.0	1.0
PCT	3/572	8339; 0.036 (0.012–0.112)	4.4 (1.3–15.0)	4.0 (1.1–14.4)
All-cause mortality				
Reference	11,756/30468	402,757; 2.919 (2.867–2.972)	1.0	1.0
PCT	150/595	8684; 1.728 (1.472–2.027)	0.4 (0.3–0.4)	0.4 (0.4–0.5)
Diseases of the liver (K70–77, B15–19, E83.1)				
Reference	735/30468	402,746; 0.182 (0.170–0.196)	1.0	1.0
PCT	4/595	8683; 0.046 (0.017–0.123)	0.2 (0.1–0.6)	0.2 (0.1–0.6)
Malignant neoplasms (C00–96)				
Reference	2323/30,468	402,746; 0.577 (0.554–0.601)	1.0	1.0
PCT	45/595	8683; 0.518 (0.387–0.694)	0.5 (0.4–0.7)	0.6 (0.4–0.8)
Drug and alcohol overdose (X40–49)				
Reference	484/30,468	402,746; 0.134 (0.134–0.157)	1.0	1.0
PCT	5/597	8.683; 0.058 (0.024–0.138)	0.5 (0.2–1.1)	0.5 (0.2–1.2)

Disease codes defined using the International Classification of Primary Care – 2nd Edition (ICPC-2) and the International Statistical Classification of Diseases 7th (ICD-7) and 10th (ICD-10) revisions. Reference group = Chronic alcohol abuse/dependence (ICD-10 = F10, ICD-9 = 303, 305.0, ICPC2 = P15). 17 persons with both PCT and a diagnosis of chronic alcohol abuse or dependence, including one person with HCC, were excluded from the analyses. 23 persons with a cancer diagnosis prior to their PCT diagnosis were excluded from any analysis in which cancer was the outcome

Mean age at death for persons with chronic alcohol abuse = 63.6 (SD: 11.7) vs. PCT = 72.5 (SD = 11.7), proportion of males = 78% vs. PCT = 50%

## Discussion

We found a 20-fold increased risk of HCC and a 7-fold increased risk of gallbladder and biliary tract cancer in persons with PCT compared to the general population. An increased risk remained also when comparing to persons with a history of chronic alcohol abuse/dependence. Further, persons with PCT had an overall 50% increased risk of premature mortality compared to the general population with 6- and 10-fold increased risks of dying from liver disease and drug or alcohol overdose, respectively.

A similar excess risk for HCC has been reported from a combined registry cohort study of 530 PCT inpatients from Sweden and Denmark [12]. Although the relative risk of HCC in our study was high when compared to the general population, the annual incidence rate (absolute risk) could be considered low at 0.07%, considering that HCC risk factors are prominent among persons with PCT. However, Norway has one of the lowest prevalence’s of HCC and chronic hepatitis B and C across Europe [21–23]. Therefore, the proportion of persons with PCT in Norway precipitated by a common HCC risk factor is likely lower than countries where such risk factors are more frequent (which is supported by the proportionally larger group of familial PCT in Norway). Consequently, it is likely that annual incidence rates of HCC in persons with PCT are higher in non-Scandinavian countries. We found in addition to HCC, a 7-fold increased risk of gallbladder and biliary tract cancer, which, to our knowledge, has only been reported once before in a case-report [24]. We also found 13 persons with PCT and lung cancer, which implied a non-significant excess risk of 60%.

Fracanzani and colleagues found that having PCT resulted in a 5-fold increased risk of HCC in 53 PCT patients compared to age, sex, liver disease severity and hepatitis C infection matched control patients with chronic liver disease [11]. However, the study may have suffered from selection bias as PCT patients were drawn from a single liver unit with a 90% prevalence of hepatitis C infection. In our population based cohort study, we found a 3-fold increased risk of HCC in persons with PCT, when compared to persons with a history of chronic alcohol abuse/dependence. Typically, alcohol abuse/dependence and liver disease, such as hepatitis C, may lead to cirrhosis and eventual HCC. Interestingly, we found that although HCC risk was greater, persons with PCT had an 80% reduced risk of dying from non-malignant liver diseases, such as viral hepatitis or liver cirrhosis, than this control group. This finding suggests that something specific to PCT may contribute to the development of HCC independent of, or by interaction with, liver disease and an excessive alcohol intake.

In line with Linet and colleagues, who reported a 70% excess risk of premature death for persons with PCT in their study [12], we found a 50% excess risk. When stratified by PCT type, however, this increased risk was only evident in persons with sporadic or unclassified PCT. We also found that having PCT resulted in a 6-fold increased risk of dying from liver diseases compared to the general population. Similarly, Linet and colleagues found increased odds of dying from cirrhosis of the liver in persons with PCT [12]. Lastly, we found an 10-fold excess risk of mortality from alcohol or drug overdose, which, to our knowledge, has not been previously reported.

### Strengths and weaknesses

Major strengths of our study are the population-based cohort design, the use of valid information from compulsory national registries, and a long period of follow-up. Persons with PCT were drawn from a nationwide registry with a good participation rate (78%). However, it is possible that participants differed from non-participants and thus selection bias may affect the validity of the results. Given the complex relationship between PCT and cancer, we chose to exclude cancer cases preceding PCT. This design reduces the risk of selection and information bias. We extend previous analyses by adjusting for education (as a proxy of socioeconomic position and lifestyle), comparing outcomes for patients with familial, sporadic and unclassified PCT, and comparing risks of PCT patients with persons with a history of alcohol abuse/dependence. A limitation of the study was the small numbers of cases. We also did not have access to liver biopsies of patients with HCC or gallbladder and biliary tract cancer. Therefore, we are uncertain regarding the proportion of these persons with concurrent fibrosis/cirrhosis.

Several common causes of PCT and cancer exist, such as alcohol use, liver disease and smoking. This information is not available in our study for the general population. Given the relationship between lifestyle factors such as excessive alcohol intake [25] and smoking habits [26] with socio-economic disadvantage and lower educational attainment, we were able to extend previous findings by controlling for educational attainment as a proxy for lifestyle.

Residual confounding by lifestyle factors is likely. We modelled residual confounding by calculating the E-value and found that an unmeasured confounder would need to have a large risk ratio of at least 39 to explain away the findings of increased HCC risk. Given the strong association between alcohol abuse/dependence and hepatitis C and both PCT and HCC, it is conceivable that unmeasured confounding may explain away any direct relationship. We were, however, able to identify persons with a history of chronic alcohol abuse/dependence to further investigate this to partially account for their generally younger age at the time of death (i.e., may have died before they could develop HCC), we conducted a competing risks survival analysis. Results, suggested persons with PCT had a 3-fold excess risk of HCC even when compared to persons with a history of alcohol abuse. The remaining high risk, albeit lower than when comparing to the general population, suggests that alcohol is an important confounder, but may not completely explain the association between PCT and HCC.

### Interpretation

It has been proposed that the accumulation of uroporpohyrins, of which levels are high in (untreated) PCT, may cause liver injury, resulting in the formation of a hepatic tumor [27]. This may occur in parallel or by interaction with exposure to hepatotoxic factors, such as alcohol. However, if the increased risk of HCC is related in part to PCT itself or is due only to confounding lifestyle factors, is difficult to conclude. Our findings give some support to the former, given that we observed this increase in the risk ratio even after controlling for educational attainment and comparing our cohort to persons with a history of chronic alcohol abuse/dependence. Of the six persons in our cohort with PCT and HCC, two had familial PCT, which is less associated with other types of liver disease or excessive alcohol intake than sporadic PCT [3]. There was a trend towards higher concentrations of total porphyrins and uroporphyrins in PCT patients with HCC compared to those without. However, the small number of cases and variability in the findings limit our ability to draw any firm conclusions. We also observed an increased risk of gallbladder and biliary tract cancer in persons with PCT. However, given we only found three cases and tested over multiple cancers, this could reflect a chance finding.

Additionally, we found an increased risk of premature death overall, death byliver diseases and drug or alcohol overdose in persons with PCT. Liver diseases, such as chronic hepatitis C virus, are known precipitating factor for PCT [10, 28], and are also associated with excessive alcohol intake and injection drug use. We also found that persons with sporadic PCT were generally more likely to die from causes associated with lifestyle factors than persons with familial PCT. Therefore, increased mortality rates in persons with PCT, and specifically sporadic PCT, could be caused by lifestyle factors.

### Clinical recommendations

According to the European Association for the Study of the Liver (EASL) and European Organization for Research and Treatment of Cancer (EORTC) clinical practice guidelines for the management of HCC, an annual incidence of 1.5% would warrant surveillance of HCC in cirrhotic patients and 0.2% would warrant surveillance in non-cirrhotic patients [29]. We found an annual incidence of HCC of 0.07% in our PCT patients, with an upper bound 95% CI of 0.17%. Additionally, unlike in acute forms of hepatic porphyria, confounding by concurrent cirrhosis cannot be ruled out in patients with PCT. Therefore, surveillance cannot be currently recommended based on a PCT diagnosis alone.

## Conclusion

We found that compared to the general population, persons with PCT had a high risk of HCC and death from alcohol or drug abuse. In addition, they had moderate excess risks of gallbladder and biliary tract cancers and of premature death in general. Although it is likely that most of our findings can be explained by common lifestyle risk factors, something inherent in PCT may contribute in part, or by interaction with lifestyle factors, to the development of HCC.

## Additional file

Additional file 1: **Table S1.** Individual characteristics of persons with hepatocellular carcinoma and gallbladder and bile duct cancer (DOCX 16 kb)
