# Supplementary material for: Porphyria cutanea tarda increases risk of hepatocellular carcinoma and premature death: a nationwide cohort study
Source: Orphanet J Rare Dis. 2019 Apr 3;14:77. doi: 10.1186/s13023-019-1051-3 (PMC6448269; doi:10.1186/s13023-019-1051-3)
Supplement: Supplementary file 1 — Table S1. Individual characteristics of persons with hepatocellular carcinoma and gallbladder and bile duct cancer (DOCX 16 kb) [file 13023_2019_1051_MOESM1_ESM.docx]

Additional file 1: Table S1. Individual characteristics of persons with hepatocellular carcinoma and gallbladder and bile duct cancer

|  | | | | | | | | | | |
| --- | --- | --- | --- | --- | --- | --- | --- | --- | --- | --- |
| Sex | Diagnosis^†^ | Topography; morphology (ICD-03)‡ | Porphyria symptoms onset – age (years) | Cancer diagnosis age (years) | Deceased at end of follow-up (Yes/no) | PCT type | Max value - total porphyrins/ uroporphyrins | HFE-status | Hemochromatosis (Yes/ no/ unclassified PCT) | Liver disease / Chronic alcohol abuse or dependence (yes/no) |
| Male | HCC | C22.0; 8170/3 | 70 | 84 | Yes | S-PCT | 369 / 234 | Very low risk | Unknown | Yes^§^ |
| Male | HCC | C22.0; 8170/3 | 62 | 74 | Yes | F-PCT | 2,073 / 1,557 | Normal | No | No |
| Male | HCC | C22.0; 8170/3 | 64 | 72 | Yes | S-PCT | 1,460 / 1,056 | Very low risk | No | No |
| Male | HCC | C22.0; 8170/3 | 54 | 66 | Yes | S-PCT | 2,172 / 1,600 | Normal | Unknown | No |
| Male | HCC | C22.0; 8170/3 | 45 | 66 | Yes | F-PCT | 171 / 118.6 | Normal | No | No |
| Male | HCC | C22.0; 8170/3 | 45 | 61 | Yes | Unknown | 150 / 60 | Not tested | Unknown | Yes^¶^ |
| Male | E-CC | C24.1 816039 | 64 | 69 | Yes | S-PCT | 2,395 / 1,619 | Very low risk | Unknown | Yes^#^ |
| Male | Ampulla of vater | C24.0 / 814039 | 50 | 62 | Yes | Unknown | 1,280 / 909 | Not tested | Yes^††^ | No |
| Female | Biliary tract | C24.9 / 816039 | 54 | 63 | Yes | S-PCT | 986 / 681 | Normal | No | No |
| Note: Disease codes defined using the International Classification of Diseases of Oncology (3rd Edition) (ICD-O3). PCT type: S-PCT=sporadic PCT, F-PCT=familial PCT. Concentration of urinary total porphyrin and uroporphyrin reflect maximum value ever recorded. Urinary porphyrins were expressed in nmol/mmol creatinine (upper reference limit < 30 nmol/mmol). HFE status refers to testing of the HFE gene variant as an indication of risk for hemochromatosis. Very low risk = “heterozygous for C282Y and negative for H63D”. ‘Hemochromatosis’ was self-reported data from the Norwegian Porphyria Registry. ‘Liver disease and alcohol chronic abuse or dependence’ registered diagnosis in the Norwegian Labour and Welfare Administration social benefit databases or the Norwegian Causes of Death Registry, including underlying and contributory causes of death. Liver disease codes defined using the International Classification of Primary Care – 2nd Edition (ICPC-2): D72 & D97; and the International Statistical Classification of Diseases 9th revision (ICD-9): 570-573 & 070 and 10th revision (ICD-10): B15-B19 & K70-K77. Disease codes defined using the ICPC-2: P15; and the ICD-9: 303, 305.0 and 10th revision (ICD-10): F10.  † HCC=hepatocellular carcinoma; E-CC=extrahepatic cholangiocarcinoma  ‡ C22.0=hepatocellular carcinoma; C24.0=extrahepatic bile duct cancer; C24.1=ampulla of vater cancer; C24.9=biliary tract cancer unclassified PCT.  § Specific ICD-10 code=K75.4 (autoimmune hepatitis)  ¶ Specific ICD-9 code=303.0 (acute alcohol intoxication) & 303.9 (other and unspecified alcohol dependence, unspecified)  # Specific ICD-10 code=K74.6 (other and unspecified cirrhosis of liver); F10 (mental and behavioural disorders due to use of alcohol) | | | | | | | | | | |
